# Immunostimulatory Properties of Dendritic Cells after *Leishmania donovani* Infection Using an *In Vitro* Model of Liver Microenvironment

**DOI:** 10.1371/journal.pntd.0000703

**Published:** 2010-06-08

**Authors:** Ludovic Donaghy, Florian Cabillic, Anne Corlu, Octavie Rostan, Olivier Toutirais, Christiane Guguen-Guillouzo, Claude Guiguen, Jean-Pierre Gangneux

**Affiliations:** 1 Université de Rennes 1, Rennes, France; 2 Inserm U522, Régulation des Equilibres Fonctionnels du Foie Normal et Pathologique, Rennes, France; 3 EE 341 Biothérapies Innovantes, Rennes, France; 4 Laboratoire de Cytogénétique et Biologie Cellulaire, CHU Rennes, Rennes, France; 5 Inserm, UMR991, Liver Metabolisms and Cancer, Rennes, France; 6 EA SeRAIC 4427, IRSET, Rennes, France; 7 Laboratoire de Parasitologie-Mycologie, CHU Rennes, Rennes, France; New York University School of Medicine, United States of America

## Abstract

**Background:**

Recent advances demonstrated that liver dendritic cells (DCs) promote immunologic hyporesponsiveness that may contribute to hepatic tolerance. Although there has been significant work on the phenotypic and functional roles of such DCs, the impact of liver microenvironment on the immune properties of infected DC is still poorly explored, probably because of the limitations of modelization.

**Methodology/Principal Findings:**

Here, we hypothesized that DC tolerogenic properties have an impact on the antimicrobial response, particularly during the infection by the protozoan parasite *Leishmania donovani.* Indeed, a lymphocytic Th2 environment was reported to favour the growth and proliferation of *L. donovani*. We first modelized an adequate monocyte-differentiated DC model, either in rat liver epithelial cell- or in a human hepatic non-parenchymal cell-conditioned medium in order to infect them further. We established that DCs differentiated in a hepatic microenvironment displayed a CD14+/CD16+/CD123+ phenotype, secreted low IL-12p70 and had an impaired capacity to stimulate allogeneic T lymphocyte proliferation and IFNγ secretion. We then infected DCs with *L. donovani* in the *in vitro*-defined hepatic microenvironment. The infection of hepatic DCs restored their capacity to stimulate allogeneic T-cell proliferation and to induce lymphocytic secretion of IFNγ. Such characteristics were recently shown to favour granuloma formation in mice liver.

**Conclusions/Significance:**

Our results suggest that the specific immunostimulatory properties of infected hepatic DCs might amplify the granuloma maturation, which warrants the effective control of infection in the liver during visceral leishmaniasis.

## Author Summary

Because of its location and function, the liver is continuously exposed to a wide range of antigens. Pathogenic microorganisms must be eliminated while a large number of dietary or commensal organism antigens and hepatic metabolites must be tolerated. Therefore, the liver has developed a specialized immune system that favours tolerance rather than immunity and liver dendritic cells (DCs) act as a major cell subtype in promoting this response. Our work aimed to examine if such immunologic hyporesponsiveness has an impact on the control of the hepatic burden of *Leishmania donovani*, a protozoan parasite that grows in liver and spleen tissues after infection (called visceral leishmaniasis in South America and Mediterranean basin, and Kala Azar in South East Asia). We first modelized an original model of hepatic DCs and infected them with *Leishmania donovani*. In contrast to control DCs, infection of hepatic DCs restored the alterate capacity of non-infected liver DCs to stimulate allogeneic T cell proliferation and IFNγ secretion. Such characteristics were recently shown to favour granuloma formation in mouse liver. This research provides an explanation for the observation that *Leishmania* parasite growth is controlled in the liver via an efficient granuloma response.

## Introduction

Parasitic infection with the obligate intracellular protozoan *Leishmania* spp. results in a broad spectrum of clinical diseases in humans. Visceral leishmaniasis (VL) is the most severe form of leishmaniasis, compared to cutaneous, diffuse cutaneous and mucocutaneous leishmaniasis. The viscerotropic species *Leishmania donovani* and *Leishmania infantum* are responsible for 500,000 new cases of VL each year in 62 endemic countries. VL ranges from asymptomatic infection, known as the sub-clinical form, to acute and potentially fatal disseminated disease with bone marrow, liver and spleen involvement [1], [2].

In the blood stream, freely circulating *Leishmania* parasites are rapidly coated by complement molecules (C3, C5…) and immunoglobulins. Complement receptors (CR1 and CR3) and Fcγ receptors expressed on macrophage and dendritic cell (DC) membranes can recognize opsonized parasites and induce phagocytosis [3], [4]. *In vitro* infection of DCs with axenic non-opsonized *Leishmania* has also indicated the involvment of the C type lectin receptor DC-SIGN (dendritic cell-specific ICAM-3-grabbing nonintegrin) in *Leishmania* internalization. DC-SIGN mediated internalization acts by recognition of mannose glycoconjugates which are strongly expressed on the cytoplasmic membrane of parasites [5], [6]. However, its precise ligand has not yet been described. DCs play a pivotal role in the control of *Leishmania* infection by directing T-cell polarization and cytokine production [3]. Their capability to modulate the immune response greatly depends on parasite diversity, with contradictory data obtained depending on whether DCs are infected with viscerotropic or dermotropic strains of *Leishmania* [7]–[15]. The response of DCs to infection also depends on their lineage, stage of maturation, processed antigens and tissue localization.

Because of its location and function, the liver is continuously exposed to a wide range of antigens. Pathogenic microorganisms must be eliminated whereas a large number of dietary or commensal organism antigens as well as hepatic metabolites must be tolerated. Therefore, the liver has developed a specialized immune system that favours tolerance rather than immunity [16]–[22]. The survival of liver allografts without the need of immunosuppressive treatment provides evidence of such tolerance [23]–[25]. Thus, hepatic DCs may play a critical role in the accurate initiation and control of the specialized immune response in the liver [18], [26]. Human liver myeloid DCs of human liver have been identified in cells spontaneously migrating from thin pieces of liver [27] and in cells eluted from donor livers prior to transplantation [28]. Human liver myeloid DCs express a semi-mature phenotype, with lower expression levels of CD80, CD86, DC-LAMP and CCR7 than those of DCs in lymph nodes [28]. Hepatic DCs express DC-SIGN, CD40 and MHC II molecules but also the common monocyte lineage marker CD14 and the plasmacytoid marker CD123 [27]. These cells synthesize IL-10 but not IL-12p70 [18], whereas IL-12p40 and IL-12p35 transcripts were detected by real-time PCR [27]. Finally, hepatic DCs stimulated T lymphocytes to secrete IL-10 and very low amounts of IFNγ [27].

One of our team has developed an *in vitro* model of monocyte-derived DCs co-cultured with rat liver epithelial cells (RLEC). This model of hepatic DCs differentiated into a CD11c^+^/CD14^+^/CD123^+^ DC subset that synthesized IL-10 but not IL-12p70 and promoted a Th2 immune response [29]. Recently, Bamboat *et al.* [30] definitely confirmed that human liver DCs promote immunologic hyporesponsiveness through the generation of suppressive T lymphocytes and a subset of IL-4 producing Th2 cells, via an IL-10-dependent mechanism.

In this study, we evaluated the molecular response of DCs to *Leishmania* infection in a hepatic microenvironment. We first developed an *in vitro* model for the differentiation of human myeloid DCs in a hepatic microenvironment, using either a rat liver epithelial cell-conditioned medium (RLEC-CM) or a human hepatic non parenchymal cell-CM (hNPC-CM). We then compared the molecular response of hepatic DCs to the infection with a hepatotropic isolate of *L. donovani*. Here, we report that DCs differentiated in a liver-CM show impaired allostimulating capacity. The infection of these DCs with *L. donovani* amplified their Th2 polarizing cytokine profile and restored their ability to stimulate the proliferation and IFNγ secretion of T lymphocytes. This response could act as a specific hepatic immunological mechanism involved in the control of parasitic infections.

## Methods

### Ethics statement

Access to the biopsy material was in agreement with French laws and satisfied the requirements of the Ethics Committee of the institution.

### Cultures of hepatic cells and production of hepatic conditioned medium

A rat liver epithelial cell line (RLEC) was isolated and cultured in William's E medium (Invitrogen, France) with 10% foetal calf serum (FCS, Invitrogen) as described by Williams *et al.* [31]. Human hepatic cells were isolated from liver biopsies by collagenase digestion [32] and centrifuged at low speed for 1 min. Hepatocytes were recovered from the pellet and the enriched hepatic nonparenchymal cell (hNPC) population from the supernatant. hNPCs were cultured in Williams' E medium with 10% FCS, 2 mM L-glutamine, 5 µg/mL bovine insulin, 5×10^−5^ M hydrocortisone hemisuccinate. When RLEC or hNPC reached confluence, the medium was removed and replaced with RPMI 1640 Glutamax-I medium (Invitrogen, France) containing 10% FCS. The 24 h culture supernatant was then recovered, filtered, and used as RLEC-CM and hNPC-CM for DC differentiation.

### Dendritic cell differentiation and maturation

Monocytes were isolated and purified from healthy blood donor buffy-coats (Etablissement Français du Sang, Rennes, France) using a RosetteSep^TM^ human monocyte enrichment cocktail (StemCell Technologies, France) and centrifugation on a Ficoll-Hypaque gradient (Sigma-Aldrich, St. Louis, Mo.). Human monocytes were then plated (2×10^6^ cells in 2 mL) into 6-well culture plates. Monocytes were cultured in RLEC-CM or hNPC-CM, both supplemented with IL-4 (500 U/mL; Peprotech Inc., Rocky Hill, NJ). Control monocytes were simultaneously cultured in RPMI 1640 Glutamax-I medium containing 25 mM HEPES, 10% FCS, IL-4 (500 U/mL) and GM-CSF (800 U/mL; Peprotech Inc., Rocky Hill, NJ). On day 2, cultures were fed by removing half of the spent medium and adding fresh medium containing cytokines to fulfill the initial volume of 2 mL. After 5 days of culture, immature myeloid DCs were harvested and recultured (5×10^5^ cells/mL) for two additional days in maturation medium containing complete culture medium supplemented with IFNγ (1000 IU/mL; Imukin, Boehringer Ingelheim, France) and *Escherichia coli* LPS (1 µg/mL; Sigma). Culture supernatant was recovered on day 7 and centrifuged prior to be cryopreserved for subsequent analysis of cytokine production. IL-10 and IL-12p70 secretions were measured using ELISA kits (optEIA; BD Biosciences, France). For the intracellular IL-12 detection, anti-IL-12 (p35/70p)-APC antibodies from Miltenyi Biotec Inc. (USA) were used according the manufacturer's recommendations.

### Parasite culture and DC infection

*L. donovani* strain MHOM/SD/97/LEM3427 Zym MON-18 was isolated from a patient with VL and was grown *in vitro* on blood agar. Prior to infection, amplification of promastigotes was carried out by culture in Schneider medium (Gibco, Invitrogen) supplemented with 10% FCS and antibiotics (penicillin 200 IU and streptomycin 200 µg/mL) for 6 days, until they reached infective stationary phase. Five-day-old DCs were then exposed to stationary phase promastigotes for 2 days. Carboxyfluorescein diacetate succinimidyl ester (CFSE, Invitrogen, France) labelling of *Leishmania donovani* promastigotes was performed at a 2 µM concentration for 10^7^ parasites/mL. Excreted-secreted antigens (ESA) from *Leishmania donovani* were obtained by centrifugation of the parasite suspension (600 g, 10 min) and 0,2 µm filtration of the supernatant.

### Flow cytometric analysis of cell surface molecules

Control and infected DCs were incubated for 30 min at 4°C with monoclonal antibodies (mAb) directed against CD14, CD16, CD40, CD86, HLA DR, CD123, DC-SIGN and CD83 (BD PharMingen, USA) and CD1c (Miltenyi Biotec, France). Isotype-matched control labelling was included in all experiments. For cell viability assays, cells were incubated 5 min with 7ADD (e-bioscience, SD, USA). Data acquisition was performed using a FACSCalibur flow cytometer and CellQuest software (Becton-Dickinson, USA). Data analyses were performed with WinMDI software (J. Trotter).

### Allogeneic stimulation assay

T lymphocytes were isolated from healthy blood donor buffy-coats with RosetteSep^TM^ human T-cell enrichment cocktail (StemCell Technologies, France) and centrifugation on Ficoll-Hypaque gradient. Mixed leukocyte reactions (MLRs) were carried out in 96-well culture plates with 200 µL of medium. Allogeneic lymphocytes (1×10^5^) were co-cultured in complete RPMI 1640 medium with 7, 5 or 3-day-old DCs differentiated either in RPMI 1640 medium or RLEC-CM and infected or not with *L. donovani*. DC∶T lymphocyte ratio of 1∶10, 1∶20 and 1∶100 were used. Lymphocyte proliferation was measured on day 6 after a 16 h pulse with [^3^H]-methyl-thymidine (1 µCi/well). CD4+ or CD8+ T lymphocyte proliferation was characterized by flow cytometry after CFSE labelling (0.2 µM concentration for 10^6^ cells/mL). To analyse cytokine production by lymphocytes, 1×10^5^ 6-day-old MLR cultures lymphocytes were restimulated with anti-CD3 (1 µg/mL) and anti-CD28 (1 µg/mL) antibodies. IFNγ, IL-4 and IL-10 secretion was measured 48 h after restimulation using ELISA kits (OptEIA, BD Biosciences).

### Statistical analysis

Statistical analyses were performed using the non-parametric Mann-Whitney test. Values of *p*<0.05 were considered statistically significant.

## Results

### Hepatic conditioned-medium influences the differentiation of monocytes in a specific hepatic DC subset

**Figure 1:**
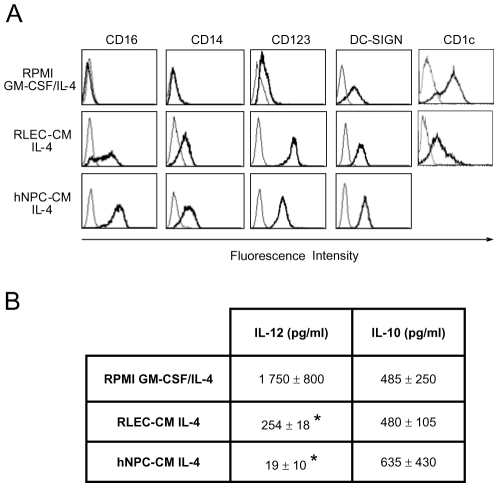
Immunophenotype and cytokine secretion profile of monocyte-derived DCs from RLEC-CM and hNPC-CM cultures. Monocytes were cultured in RPMI medium, RLEC-CM or hNPC-CM with the indicated cytokine combinations. On day 5, DCs were harvested and recultured (5×10^5^ cells/ml) for 2 days in the presence of IFNγ (1000 IU/ml) plus LPS (1 µg/ml). **A.** Cells were stained with specific antibodies (thick line histograms) or isotype controls (thin line histograms). Results for a single representative donor out of at least three donors are shown. **B.** Production of IL-10 and IL-12p70 was measured by ELISA after 48 h of maturation. Results are expressed as the mean ± SD of at least three experiments. When indicated, the mean value was statistically significant compared to control DCs (*, *p*<0.05).

The influence of the hepatic environment on human monocyte differentiation was investigated using RLEC and hNPC conditioned-medium (CM). Monocytes cultured in both RLEC-CM and hNPC-CM differentiated into a CD16+/CD14+/CD123+ DC subset as shown in figure 1A. By contrast, control DCs were CD16-/CD14-/CD123low. Over-expression of co-stimulatory (CD40 and CD86) and MHC-class II molecules was observed (data not shown) as previously shown using a co-culture model [29]. Regarding the cytokine secretion, mature RLEC-CM- and hNPC-CM-differentiated DCs produced IL-10 at similar level than control DCs, but IL-12p70 secretion was dramatically reduced compared with control DCs (*p*<0.05) (Fig. 1B). Thus, both RLEC-CM and hNPC-CM DCs display the same specific hepatic phenotype and secretion profile.

### RLEC-CM DCs have an impaired capacity to stimulate allogeneic T lymphocyte proliferation

**Figure 2:**
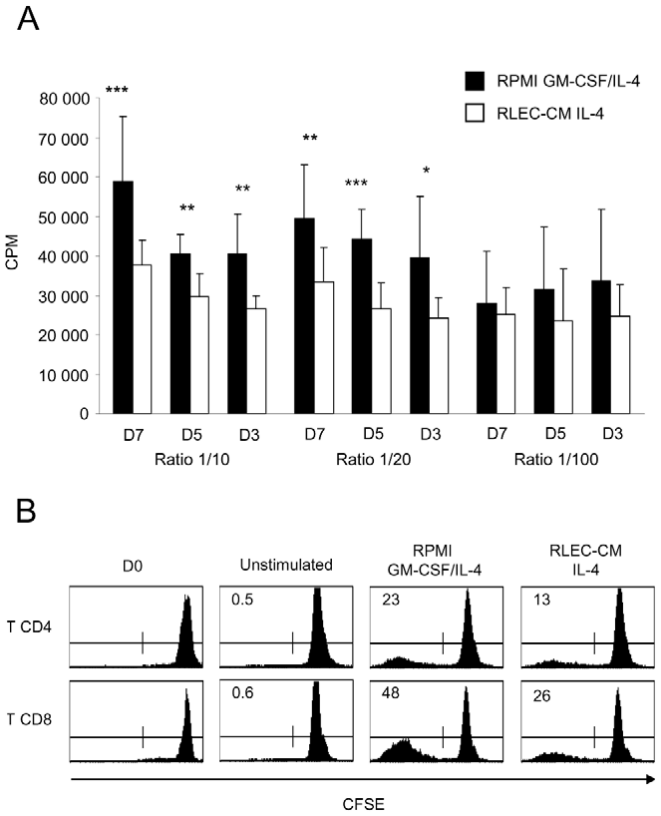
Allogeneic lymphocyte proliferation induced by DCs from RLEC-CM culture. DCs were differentiated and matured (IFNγ/LPS) in RPMI medium in the presence of GM-CSF and IL-4, or in RLEC-CM with IL-4 and then cultured for 6 days with allogeneic T lymphocytes (1×10^5^). **A.** Lymphocyte proliferation induced by 3, 5 and 7-day-old DCs was measured after a 16 h pulse with [^3^H]-methylthymidine (1 µCi/well); DC∶T lymphocyte ratios of 1∶10, 1∶20 and 1∶100 were used. *, *p*<0.05; **, *p*<0.01; ***, *p*<0.001. **B.** T lymphocytes were labelled with CFSE and stained with anti-CD4 and anti-CD8 antibodies for flow cytometry analysis. Percentage of proliferating cells is indicated on the histograms.

To determine the capacity of this hepatic DC subset to induce an immune response, we carried out MLRs. Allogeneic T lymphocytes were co-cultured with 7, 5 ou 3 day-old DCs. Thymidine incorporation assays demonstrated an impaired capacity of RLEC-CM DCs to induce allogeneic lymphocyte proliferation for both 1∶10 and 1∶20 DC∶T lymphocyte ratios, compared with control DCs (Fig. 2A). Flow cytometry analysis of CFSE-labelled lymphocytes showed that the percentage of proliferating T lymphocytes was two-fold lower with RLEC-CM DCs than control DCs (13% vs 23% and 26% vs 48% for T CD4 and CD8, respectively). Moreover, the CD4∶CD8 proliferating T lymphocyte ratio was similar *i.e.* 1∶2 for both RLEC-CM and control DCs (Fig. 2B).

### *L. donovani* infection does not alter the phenotypic maturation of RLEC-CM DCs

**Figure 3:**
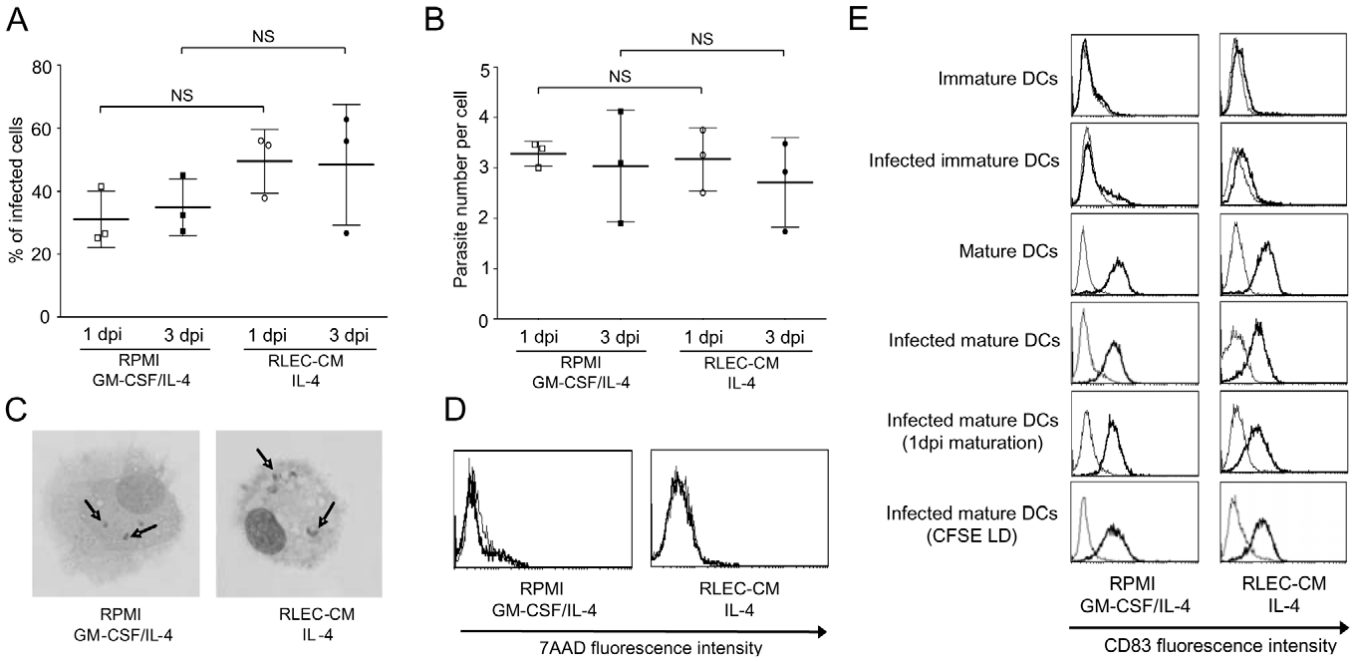
Infection of RLEC-CM and control DCs by *L. donovani*. Monocytes were cultured in RPMI or RLEC-CM with the indicated cytokine combinations. On day 5, DCs were harvested and recultured (5×10^5^ cells/ml) for 2 days in the presence of IFNγ (1000 IU/ml) plus LPS (1 µg/ml) with or without *L. donovani* parasites (10∶1). **A/B.** Percentage of infected cells and parasite number per infected cell: one and three day post-infection (dpi) cells were analysed on May-Grünwald-Giemsa-stained cytospins. Data are the percentage of infected cell number/total cell number and the parasite number per infected cells; data are mean ± SD from three experiments; 250 cells were counted per experiment; NS = not significant difference. **C.** Photographs of May-Grünwald-Giemsa-stained cytospins (arrows indicate intracellular parasites). **D.** Cell viability: cells were stained with 7ADD (thick line histograms) or unstained (thin line histograms). Results for a single representative donor out of three donors are shown. **E.** CD83 expression: cells were stained with anti-CD83 antibody (thick line histograms) or isotype control (thin line histograms). In some experiments, maturation was performed one day post-infection (1 dpi maturation). When CFSE-stained *L. donovani* promastigotes (CFSE LD) were used, analysis of CD83 expression was realized on CFSE-positive cells.

To assess the effects of *L. donovani* infection on the immunophenotype and cytology of RLEC-CM DCs, infection with *L. donovani* was carried out during the final 2 days of DC culture. The percentage of infected DCs ranged between 31% and 35% for control DCs and was 49% and 48% for RLEC-CM DCs at days 1 and 3, respectively (Fig. 3A). The average of intracellular amastigote number was of 3 per infected cells, either in RLEC-CM and control DCs. This level of infection remained stable between day 1 and day 3 suggesting their intracellular survival (Fig. 3B and 3C). The viability of infected DCs was preserved as shown by staining with the marker of cell viability 7AAD (Fig. 3D). In addition, the infection of immature DCs did not induce the CD83 maturation marker expression (Fig. 3E). By contrast, LPS and IFNγ maturation stimuli induced CD83 expression in non-infected as well as in infected RLEC-CM and control DCs (Fig. 3E). Of note that the use of CFSE-labelled parasites evidenced that the up-regulation of CD83 was similar in the infected cell and non infected cell populations (Fig. 3E). Moreover, the immunophenotype (CD16, CD14, CD123, DC-SIGN, CD40, CD86 and HLA-DR) of infected mature RLEC-CM and control DCs was similar to the non-infected mature cell phenotype (data not shown).

### *L. donovani* infection modifies the cytokine secretion profile of mature RLEC-CM DCs

**Figure 4:**
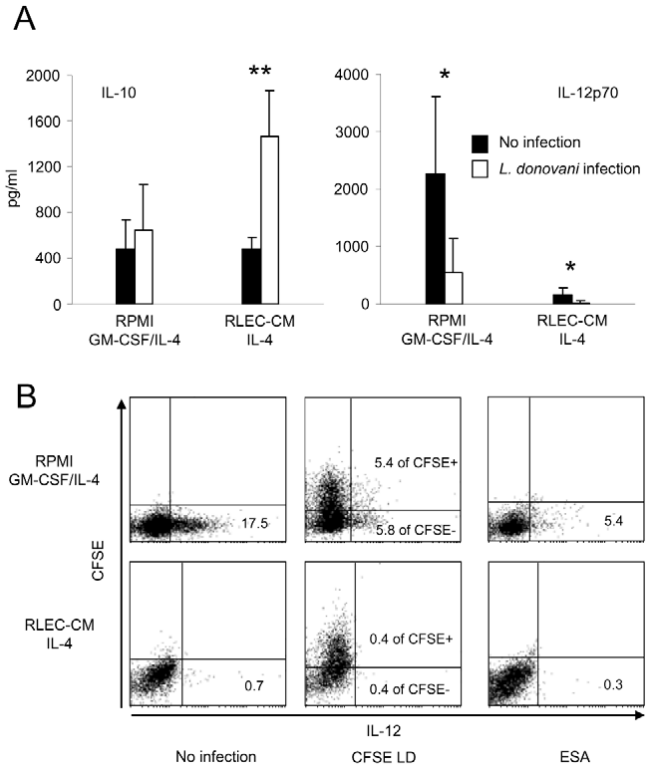
Cytokine secretion profile of DCs after *L. donovani* infection. Monocytes were cultured in RPMI medium or RLEC-CM with the indicated cytokine combinations. On day 5, DCs were harvested and recultured (5×10^5^ cells/mL) for 2 days with IFNγ (1000 IU/mL) plus LPS (1 µg/mL). **A.** *L. donovani* infection was carried out at a ratio of 10∶1 (parasites:DCs). Secretion of IL-10 and IL-12p70 was measured in the culture supernatant by ELISA after 48 h. Results are expressed as the mean ± SD of at least three experiments. When indicated, the mean value was statistically significant compared to non infected DCs (*, *p*<0.05; **, *p*<0.01). **B.** Infection was carried out with CFSE-labelled parasites or excreted-secreted antigens (ESA) from *L. donovani*. Intracellular staining of IL-12 was evaluated by flow cytometry analysis. Percentage of IL-12 positive-cells among the whole cell population or among infected cells (CFSE+) and non infected cells (CFSE-) is indicated on the cytograms.

IL-10 and IL-12p70 secretion was measured in culture supernatants of 7-day-old infected mature RLEC-CM and control DCs. *L. donovani* infection did not modify control DC secretion of IL-10 (Fig. 4A). Conversely, infection of RLEC-CM DCs induced a statistically significant up-regulation of IL-10 secretion (*p*<0.01). IL-12p70 secretion was markedly higher in non-infected mature control DC supernatants than in non-infected RLEC-CM-differentiated DC supernatants. Infection of both RLEC-CM and control DCs by *L. donovani* resulted in significant decreased IL-12p70 levels (*p*<0.05) (Fig. 4A). IL-12 intracellular staining of DCs infected with CFSE-labelled parasites or excreted-secreted antigens from parasites evidenced that secretion ability was similarly reduced in infected and non-infected cell populations (Fig. 4B).

### *L. donovani* infection restores the allostimulating capacity of mature RLEC-CM DCs

**Figure 5:**
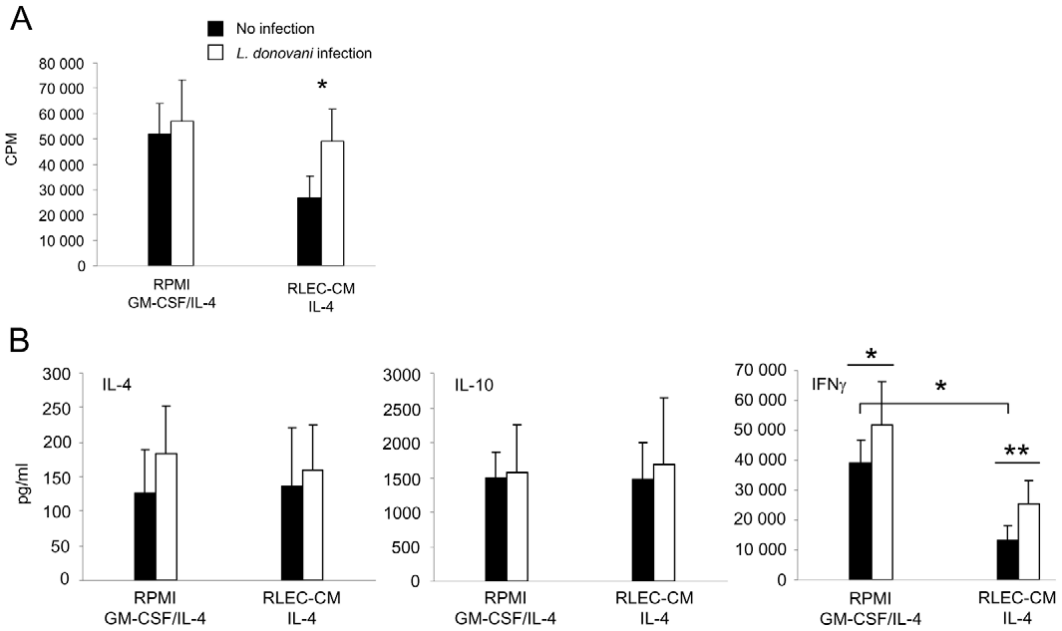
Proliferation and cytokine secretion of allogeneic lymphocytes stimulated with DCs after *L. donovani* infection. DCs were differentiated and matured (IFNγ/LPS) in RPMI medium in the presence of GM-CSF and IL-4, or in RLEC-CM with IL-4. Five-day-old DCs were infected or not with *L. donovani* (10∶1). Allogeneic lymphocytes (1×10^5^) were co-cultured with 7-day-old mature infected or non-infected DCs (5×10^3^). **A.** Lymphocyte proliferation was measured on day 6 after a 16 h pulse with [^3^H]-methylthymidine (1 µCi/well). **B.** Lymphocytes were collected after 6 days of stimulation, washed and restimulated for 48 h with anti-CD3 and anti-CD28 mAbs. Supernatants were harvested and analysed by ELISA for IL-4, IL-10 and IFNγ secretion. Results are expressed as the mean ± SD and represent at least three experiments. When indicated, the mean value is statistically significant compared to non-infected DCs (*, *p*<0.05; **, *p*<0.01).

MLRs were carried out in order to determine the impact of *L. donovani* infection on the capacity of RLEC-CM and control DCs to stimulate allogeneic T lymphocyte proliferation and cytokine secretion. *L. donovani* infection of control DCs did not modify allogeneic lymphocyte proliferation (Fig. 5A). In contrast, infection of mature RLEC-CM DCs significantly restored their capacity to activate allogeneic lymphocyte proliferation (*p*<0.05). Regarding the cytokine secretion, lymphocytes primed with mature RLEC-CM DCs, and subsequently restimulated with anti-CD3 and anti-CD28 mAbs, secreted significantly less IFNγ than those primed with control DCs (*p*<0.05) (Fig. 5B). The IFNγ/IL-10 and IFNγ/IL-4 ratios from RLEC-CM DCs were lower than those of control DCs (9 vs. 26 and 96 vs. 307, respectively). Interestingly, lymphocytes stimulated with mature *L. donovani* infected RLEC-CM DCs, as well as with mature infected control DCs, secreted significantly higher amounts of IFNγ compared to non-infected DCs (*p*<0.01 and *p*<0.05, respectively) (Fig. 5B).

## Discussion

The hepatic DC response to hepatotropic pathogens is still poorly understood probably because only limited models of hepatic DCs are available [18]. Human liver DCs have been isolated and identified in cells eluted from donor livers prior to transplantation [28] and in freshly purified cells from thin pieces of liver [27], [30]. Analyses of such *ex vivo* hepatic DCs revealed a semi-mature phenotype [28] and expression of membrane markers commonly associated with other lineages such as CD14 and CD123 [27]. These liver DCs induced less alloproliferation and promoted T cell hyporesponsiveness [30]. *In vitro* hepatic models were also recently developed to generate human monocyte-derived DCs in co-culture with either RLECs [29] or mouse liver fibroblastic stromal cells [21]. *Ex vivo* and *in vitro* co-cultured hepatic DCs failed to synthesize IL-12p70 and promoted a Th2 immune response orientation [18], [21], [27], [29], [30]. All together, these results suggest that liver DCs have tolerogenic properties and put into question the impact of such characteristics on the antimicrobial response.

Due to the presence of feeder cells [21], [29] or a relatively low purification levels [26], these models are not designed for infection protocols. Cabillic *et al*. [29] suggested that hepatic characteristics of DCs could be related to soluble molecules such as cytokines and growth factors produced by the local environment. We then developed a model of human monocyte-derived DCs in a hepatic microenvironment without feeder cells using conditioned medium from either hNPCs and RLEC. Here, we showed that DCs generated in both hepatic cell-conditioned media share features with control DCs, including typical morphology and expression of DC-SIGN as well as over-expression of MHC II (HLA-DR) and co-stimulation molecules (CD40, CD86), compared to monocytes. DCs differentiated in hNPC- and RLEC-CM display a similar immunophenotype defined as a CD14+/CD123+ DC subset, in accordance with previous studies using other experimental models [27], [29], [33]. Furthermore, we showed that CD123 expression was increased during maturation of these cells and that they also expressed CD16. Mature control DCs secreted IL-10 and IL-12p70 and activated allogeneic lymphocyte proliferation. Contrastingly, DCs differentiated in both hepatic microenvironments secreted very low amounts of IL-12p70, were poor inducers of lymphocyte proliferation, as described *ex vivo* by Goddard *et al.* [27]. In accordance with *ex vivo* [27], [30], [34], [35] and *in vitro* [21], [29] studies, our results confirm that liver constitutes a specific micro-environment which contributes to the tolerogenic properties of DCs.

We then analysed the molecular response of this DC subset to parasitic infection. Because primary human hNPCs were isolated from human liver biopsies, it was easier to use an *in vitro* model with RLEC-CM for infection protocols. We selected the hepatotropic protozoan *L. donovani* and examined the impact of an hepatic microenvironment on DC response to infection. Whereas recent studies with dermotropic *Leishmania* strains highlighted the importance of a dermal cytokine microenvironment for initiation of a local inflammatory response [36], no data are available concerning the liver, one of the main target organ during VL. Here we showed that *L. donovani* infection did not induce the maturation of DCs when infecting either immature DCs differentiated in control medium or in RLEC-CM. Indeed, we neither observed CD83 expression nor over-expression of co-stimulatory molecules CD40 and CD86 or HLA-DR, and there was no induction of IL-12p70 secretion (data not shown). Our results are in agreement with those obtained with *L. mexicana* and *L. amazonensis* suggesting that the promastigote surface is devoid of DC-activating signals and that DCs infected with stationary-phase promastigotes remain phenotypically immature [8], [13]. In addition, we showed that *L. donovani* parasite did not further inhibit the maturation process of infected DCs (i.e., over-expression of co-stimulatory molecules and MHC II, expression of CD83 and morphological modifications). However, in contrast with the findings from McDowell *et al*. [11], we observed a strong inhibition of IL-12p70 secretion during infection of mature RLEC-CM and control DCs.

In our experimental model, *L. donovani* infection increased the IL-10 secretion of DCs differentiated in RLEC-CM. Thus, infected hepatic DCs may be partly responsible for the high serum IL-10 levels observed in patients with active VL [37], [38]. Furthermore, *L. donovani* infection of DCs differentiated in RLEC-CM restored their impaired capacity to stimulate allogeneic lymphocyte proliferation and up-regulated lymphocyte IFNγ secretion. A similar simultaneous co-expression of IL-10 and IFNγ is detected in the serum of acute VL patients [37], [38]. Furthermore, a *Leishmania*-specific subtype of CD4+ lymphocytes from individuals cured of VL has even been described as IFNγ+/IL-10+ [39]. In this context, Amprey *et al*. [40] have described a liver-specific secretion of IFNγ a few hours after *Leishmania* infection. This secretion was independent of IL-12p70 and was suggested to be produced by a subset of NKT cells.

In the liver, parasites are phagocytosed by Küpffer cells and DCs. Infected cells secrete cytokines and recruit lymphocyte sub-populations in order to create a granuloma. A well-formed mature granuloma is an antigen-specific immune response mediated by mononuclear cells. Such mature granuloma presents a delayed appearance but a long-term persistence. During human VL, mature hepatic granulomas commonly correlate with infection control and clinical latency [41]. Cytokines such as IFNγ and TNFα are involved in the activation of immunocompetent cells and account for the final microbicidal response [1], [41]. However, variable responses can be observed, depending on the host's genetic and immunological environment. Using a non-infected model of IL-1 receptor antagonist-deficient mice, impaired DC maturation associated with IL-10 secretion was shown to exacerbate granuloma formation in murine liver [42]. Moreover, the transfer of liver DCs loaded with an antigen to the portal vein of mice was shown to induce a Th2 orientation of immune response [43]. During *Schistosoma mansoni*/*L. donovani* co-infection, Hassan *et al*. [44] similarly demonstrated that mature hepatic granuloma formation was favoured by the Th2 cytokine environment induced by *S. mansoni* infection. All together, an efficient granuloma response in a Th2 environment may contribute to control of infection. Hepatic DCs may then represent a major cell subtype involved in the induction and maintenance of such Th2 environment. This newly described *in situ* activation of the liver immune response might represent a complementary phenomenon with the model proposed by Engwerda *et al*. [45], [46], in which lymphocytes are primed in the spleen, where IL-12p70 production is important, and then migrate to the liver. Our data show that *L. donovani* infection of DCs within a hepatic environment restores their ability to induce allogeneic lymphocyte proliferation and IFNγ secretion. Such results suggest the existence of an intrinsic liver activation pathway that could contribute to granuloma formation, in addition to the spleen-mediated activation way described previously.
